# Enzyme Therapy: Current Challenges and Future Perspectives

**DOI:** 10.3390/ijms22179181

**Published:** 2021-08-25

**Authors:** Miguel de la Fuente, Laura Lombardero, Alfonso Gómez-González, Cristina Solari, Iñigo Angulo-Barturen, Arantxa Acera, Elena Vecino, Egoitz Astigarraga, Gabriel Barreda-Gómez

**Affiliations:** 1 Department of Research and Development, IMG Pharma Biotech S.L., 48160 Derio, Spain; miguel@imgpharma.com (M.d.l.F.); laura@imgpharma.com (L.L.); egoitz.astigarraga@imgpharma.com (E.A.); 2 Experimental Ophthalmo-Biology Group, Department of Cell Biology and Histology, University of the Basque Country UPV/EHU, 48940 Leioa, Spain; aacera71@gmail.com (A.A.); elena.vecino@ehu.eus (E.V.); 3 Department of Molecular Life Sciences, University of Zurich, Winterthurerstrasse 190, CH-8057 Zurich, Switzerland; alfonso.gomezgonzalez@uzh.ch; 4 Department of Pharmacology and Toxicology, University of Zurich, Winterthurerstrasse 190, CH-8057 Zurich, Switzerland; cristina.solari@uzh.ch; 5 The Art of Discovery, 48160 Derio, Spain; inigo.ab@tad-med.com

**Keywords:** biotechnology, enzyme therapy, encapsulation, molecular modification of enzymes, monitoring of immune response, microarray, COVID-19

## Abstract

In recent years, enzymes have risen as promising therapeutic tools for different pathologies, from metabolic deficiencies, such as fibrosis conditions, ocular pathologies or joint problems, to cancer or cardiovascular diseases. Treatments based on the catalytic activity of enzymes are able to convert a wide range of target molecules to restore the correct physiological metabolism. These treatments present several advantages compared to established therapeutic approaches thanks to their affinity and specificity properties. However, enzymes present some challenges, such as short in vivo half-life, lack of targeted action and, in particular, patient immune system reaction against the enzyme. For this reason, it is important to monitor serum immune response during treatment. This can be achieved by conventional techniques (ELISA) but also by new promising tools such as microarrays. These assays have gained popularity due to their high-throughput analysis capacity, their simplicity, and their potential to monitor the immune response of patients during enzyme therapies. In this growing field, research is still ongoing to solve current health problems such as COVID-19. Currently, promising therapeutic alternatives using the angiotensin-converting enzyme 2 (ACE2) are being studied to treat COVID-19.

## 1. Introduction

Enzymes are chemical catalysts of biological systems. They allow organisms to self-replicate and catalyze, in a selective and efficient manner, essential biochemical reactions. Enzymes are proteins, except for ribozymes, which are a small group of RNA molecules with a catalytic activity [1]. These proteins have a high specificity that allows them to discriminate between substrates with similar structures [2]. Furthermore, they possess an extraordinary catalytic power that accelerates the targeted chemical reactions. The process of catalyzing biochemical reactions takes place in aqueous solutions under very mild conditions of temperature and pH [3].

Enzymes are essential in biochemical processes. They catalyze hundreds of stepwise metabolism reactions, preserving and transforming chemical energy and generating biological macromolecules from precursors. Their catalytic activity depends on the integrity of their native protein conformation. In this regard, the activity of one or more enzymes is impaired in many diseases due to mutations [4]. Because of the necessity of the correct performance of the enzymes, many drugs have been developed with the aim to target dysfunctional enzymes [5].

An alternative approach is to use enzymes directly as therapeutic drugs. They were used firstly at the end of the 19th century, when enzymes such as pepsin were used to treat dyspepsia [6].

In 1987, the first recombinant enzyme drug for acute ischemic stroke, plasminogen activator Alteplase, was approved by the Food and Drug Administration (FDA, Montgomery, MD, USA) [7]. This drug was prescribed for the treatment of acute ischemic stroke thanks to its capacity to dissolve clots and restore tissue perfusion [8]. To support the growing demand for these enzymatic treatments, major efforts are being invested in their industrial production, using recombinant expression of these molecules in plants, mammalian systems and microbial systems (fungi, yeast or bacteria) [9]. However, some enzyme drugs are taken directly from nature, for instance, snake venom [10,11].

**Figure 1:**
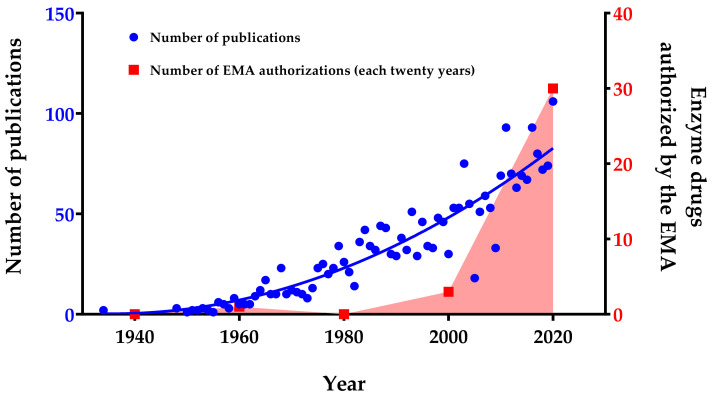
Number of publications and enzyme drugs authorized per year (from 1934 to 2020). Publication searches were performed by entering the subject “enzyme therapy, drug and treatment” in PubMed database and choosing the field “Title/Abstract” to filter the search. The enzyme drug searches were performed in the European Medicines Agency (EMA, Amsterdam, The Netherlands) database, and the number of authorized enzymes per twenty-years’ time intervals was plotted. Red area only highlights the growing trend in the number of enzymes authorized by the EMA.

The industrial market for enzyme-based drugs is expected to increase at a compound annual growth rate of 6.8% within the period 2019–2024 [12]. In 2024, markets that involve proteases or carbohydrase are estimated to reach 2 and 2.5 billion USD, respectively [13]. These market indicators are reflected in an increase in the number of enzyme drugs authorized in recent years (Figure 1). Together with this economic growth, an increase in the number of publications concerning enzyme therapy has been observed, highlighting the growing interest and potential of this field (Figure 1). The observed increase in research publications and patents to date highlights the efforts invested in this field mainly because of the promising therapeutic potential of enzymes. Presently, enzymes are not only being used and investigated for the treatment of metabolic deficiencies but also for many different pathologies such as cancer and cardiovascular diseases [14,15,16,17].

The potential of enzyme-based drugs can be improved in regard to specific factors. First, the in vivo half-life of the molecules should be improved; second, the targeted action is not always accurate; and third, valid methods are necessary to control the patient’s immune system response during treatments based on enzymes [18]. In this context, novel approaches to monitor the immune response, such as microarrays, are of ongoing interest for personalized medicine. Moreover, newer approaches based on enzymes are being studied to treat infections such as SARS-CoV-2 and its associated pathology, COVID-19, highlighting the potential benefit of enzyme therapy.

The aim of this review is to approach the field of enzyme therapy from another perspective, which it integrates not only treatment examples, but also their current challenges, as well as new trouble-solving strategies. This contribution confers a new and broader vision of the area of enzyme therapeutics.

## 2. Methodology

In the present review, a systematic search of recent literature was performed. The used databases were: PubMed, Google Scholar, Web of Science and ScienceDirect; with a particular attention to recent publications (<5 years). The performed search contained the words: “enzyme therapy”, “enzyme drug”, and “enzyme treatment”. The main focus concerned the description of the principal pathologies treated with enzymes, the targeted pathways and the used enzymes, as well as the main problems and advantages of these types of therapies. The different uses of therapeutic enzymes were obtained from the drug database of the EMA (https://www.ema.europa.eu/en/medicines, accessed on 13 July 2021), using the anatomical therapeutic chemical (ATC) index as search criteria. ATC numbers are included in the References section.

## 3. Enzyme Therapies for Different Pathologies

Since their first uses as drugs, enzymes have been widely applied to treat enzymatic deficiencies and several health issues.

Therapies based on enzymes can be systemic or non-systemic, and they have multiple administration routes: oral [19], topic [20], respiratory [21] or intravenous [22]. We classified the main pathologies treated with enzymes according to the type of disease. A summary of the categorization is included in the table at the end of this section (Table 1), which will also be referenced in the subsections for each type of disease.

### 3.1. Metabolic Deficiencies

Pathologies caused by the absence or deficiency of an enzyme are the main targets for enzyme replacement therapy (ERT). These medical treatments are employed to try to restore the lost or altered enzymatic activity. Usually, the enzyme is administrated through an intravenous solution. The main metabolic deficiencies treated with ERT are the lysosomal storage diseases (LSD).

#### 3.1.1. Lysosomal Storage Diseases (LSD)

LSD are a heterogeneous group of rare inherited metabolic disorders that are the result of lysosomal dysfunctions. They originate from a deposit of noncatalyzed glycosaminoglycans, which is caused by a deficiency in lysosomal enzymes or alterations in molecular transport. Gaucher’s disease, Hunter’s syndrome, Fabry’s disease, Hurler’s syndrome, Morquio syndrome type A, Maroteaux-Lamy syndrome, Sly syndrome, α-mannosidosis, Batten disease and Pompe’s disease are examples of disorders included in the LSD group. At the moment, some biomarker discovery projects are underway to improve LSD diagnosis [23,24]. Due to the features of the above-mentioned pathologies, ERT appears to be a promising therapeutic alternative. A summary of LSD treated with enzyme drugs is showed in Table 1.

Gaucher’s disease is caused by the loss of the glucocerebrosidase enzyme, which leads to the accumulation of lipids, such as glucocerebroside, especially in the bone marrow, spleen and liver. As a consequence, swollen liver and/or spleen, anemia, thrombocytopenia and skeletal abnormalities can be present in affected patients. In this context, ERT is able to balance the low levels of glucocerebrosidase with the administration of a recombinant version of the enzyme through intravenous injections [25].

Hunter’s syndrome, also known as Mucopolysaccharidosis type II, is a rare and inherited pathology triggered by the deficiency of iduronate 2-sulfatase (I2S), an enzyme catalyzing the degradation of the glycosaminoglycans dermatan- and heparan-sulfate. In the absence of these enzymes, molecules accumulate in organs and tissues, leading to an imbalance in normal homeostasis that can influence physical and mental development. In these cases, recombinant I2S is administrated intravenously as an ERT, leading to improvement of the clinical parameters [26].

Fabry’s disease is a rare and inherited condition triggered by a deficiency of the lysosomal enzyme α-galactosidase A (AGAL). Thus, a progressive deposition of an incomplete metabolized lipid substrate (Gb3) is observed in multiple cell types, causing alterations in vascular reactivity and a propensity for thrombo-embolic disease [27]. These abnormalities are believed to play a role in increased risk for particular problems, with renal and cardiac failure being the main causes of morbidity [27]. An intravenous infusion of a recombinant form of AGAL as ERT can improve the course of the disease [28].

Hurler’s syndrome, Morquio syndrome type A, Maroteaux-Lamy syndrome, Sly syndrome, α-mannosidosis, Batten disease and Pompe’s disease are other examples of LSD, characterized by α-L-iduronidase, N-acetylgalactosamine-6-sulfate sulfatase, arylsulfatase B, β-glucuronidase, α-D-mannosidase, tripeptidyl peptidase 1 and acid α-glucosidase deficits, respectively. To treat these pathologies, ERT represents the best therapeutic approach [29,30,31,32,33,34,35].

#### 3.1.2. Further Metabolic Deficiencies

In addition to LSD, there are several other metabolic deficiencies that need to be considered (Table 1).

Exocrine pancreatic insufficiency (EPI) is characterized by an impaired secretion of pancreatic enzymes and bicarbonate. EPI can be caused by upper gastrointestinal and pancreatic surgery as well as by different pancreatic diseases, such as cystic fibrosis (CF). The consequent maldigestion and malabsorption of nutrients leads to several nutritional deficiencies. To improve patients’ quality of life, pancreatic ERT represents a valid approach [36,37]. However, nutrient malabsorption has also been observed in acquired immunodeficiency syndrome (AIDS), whose related experimental studies have shown promising results for the use of pancreatic ERT in the improvement of this condition [38].

Phenylketonuria (PKU) is an inborn disease caused by mutations in the phenylalanine hydroxylase (PHA) gene. These alterations lead to an enzyme deficiency that causes hyperphenylalaninemia. One of the approaches to control phenylalanine concentration is to use a PHA ERT. For this purpose, unmodified PHA and phenylalanine ammonia-lyase PHA can be administrated [39].

Severe combined immunodeficiency (SCID) is a group of rare pathologies, in which the genes involved in the development and function of immune cells are mutated. One subtype of SCID is characterized by adenosine deaminase (ADA) enzyme deficiency. The function of this enzyme is necessary for the breakdown of adenosine absorbed from food and for the turnover of nucleic acids in tissues. Its insufficiency leads to the accumulation of toxic purine degradation products, which mostly affect lymphocytes, causing immunodeficiency. ERT based on polyethylene glycol-conjugated adenosine deaminase (PEG-ADA) shows an improved life quality [40]. PEG modifications reduce the plasma clearance of the enzyme, as they decrease cellular uptake, proteolysis and immunogenicity compared to the unmodified enzyme. As a consequence, circulating levels and the in vivo half-life of the therapeutic enzyme are improved [41].

Many other metabolic diseases in which ERT can play a crucial role are mentioned below. Wolman disease, which is characterized by the absence of the lysosomal acid lipase (LAL) enzyme, could be treated by administrating LAL as an ERT [42]. In acute intermittent porphyria (AIP), the deficiency of the enzyme hydroxymethylbilane synthase (HMBS), also known as porphobilinogen deaminase (PBGD), could be addressed by administrating an ERT based on HMBS/PBGD [43]. Furthermore, congenital sucrase-isomaltase (SI) deficiency (CSID) is the result of a reduction or loss of the SI enzyme, which could be treated with an ERT by administrating Sucraid (sacrosidase) [44]. In cases with hypophosphatasia, which is a disease characterized by the tissue-nonspecific isoenzyme of alkaline phosphatase (TNSALP) deficiency, TNSALP ERT represents a valid treatment [45]. Protein C deficiency can also be treated with ERT by administering the protein [46]. Lastly, ERT can also be used in cases of lactase deficiency by delivering microbial recombinant lactase [47].

### 3.2. Fibrosis Conditions

Interest in peptidase enzymes is increasing due to their capacity to degrade protein deposits in different types of tissues. Metalloprotease endopeptidases, which include collagenases and gelatinases (such as matrix metallopeptidase, MMP, 9 or 2), are being studied as treatments for different pathologies. Table 1 presents a synopsis of the different fibrosis conditions treated with enzymes.

Chronic total occlusion (CTO) is a complete or partially complete obstruction that concerns coronary arteries. The blockage is produced by the accumulation of a collagen plaque in a coronary artery, which could compromise blood flow to the heart. One of the current therapies is the local administration by catheter of type IA collagenase, a bacterial collagenase formulation obtained from Clostridium histolyticum (CCH, Collagenase Clostridium histolyticum) which is able to degrade the collagen plaques [48]. Furthermore, CCH is administrated also in Dupuytren’s disease for the enzymatic removal of the fibrotic fascia (fasciotomy). This pathology is characterized by the thickening of the fascia, which is the fibrous layer of tissue that lies underneath the skin of the palm and fingers. As a result of this abnormality, hands present some deformations [22,49]. Lastly, CCH is also applied for the enzymatic digestion of fiber plaques and fiber tissue found in Peyronie’s disease [20] and Uterine fibroids [50], respectively.

Keloids, lung CF and glaucoma are further examples of fibrosis conditions that can be treated with enzymes. Keloids are fibroproliferative dermal tumors with effusive accumulation of extracellular matrix that can generate after surgery. Collagenases and matrix metallopeptidases have been demonstrated to be safe and efficient in reducing keloids [51,52]. Moreover, lung CF is a pathology caused by the formation of thickened mucus in the lungs. A recombinant form of deoxyribonuclease I (Dornase α) can be administrated to dissolve the secretions [53]. Glaucoma represents a group of eye conditions that damage the optic nerve, leading to diverse vision problems, and are potentially able to cause blindness. In many cases, fibrosis is known to occur as a consequence of extracellular matrix accumulation in the trabecular meshwork at the anterior part of the eye and in the lamina cribrosa at the optic nerve head. A novel method to reduce fibrosis through administration of purified collagenase into a patient eyes has been patented [54].

### 3.3. Ocular Affections

Retinal detachment, macular pucker, diabetic retinopathy, macular holes, vitreous hemorrhage and vitreous floaters are ocular pathologies that can be treated with a vitrectomy, which is a surgery to remove some or all the vitreous humor of the eye. However, the use of enzymes, such as chondroitinase, hyaluronidase, nattokinase or ocriplasmin, allows the non-invasive removal of the vitreous humor simply by digestion [55] (Table 1).

### 3.4. Joint Problems

Different conditions related to chronic and pathological joint problems, associated with pain and inflammation, are being treated with enzymes (Table 1).

Intradural disc herniation (IDH) occurs when disc material penetrates the spinal dura and lies in an extramedullary location. IDH can be treated with chemonucleolysis by injecting an enzyme into the vertebral disc, aiming to dissolve its inner part. Sulfate ABC endolyase, an enzyme that catalyzes the depolymerization of chondroitin sulfate, is used for this purpose [56].

Arthritis, especially osteoarthritis and rheumatoid arthritis, is a pathology that causes pain and inflammation in a joint. Anti-inflammatory drugs, combined with proteolytic enzyme supplements, show diminished pain and improved quality of life [57,58].

### 3.5. Cancer

Cancer has one of the highest incidences in the world, and therefore, great efforts are taken to find successful therapies. In this sense, it could be said that many of the therapeutic strategies under study are based on enzymes (Table 1).

When the tumor microenvironment is characterized by an elevated amino acid metabolism, which is required for cancer cells to grow, proliferate and survive, different enzymes targeting these molecules become attractive therapeutic alternatives. PEGylated arginine deaminase has been approved and PEGylated kynureninase is currently under study to deal with increased arginine and tryptophan presence in tumor microenvironment, respectively. The latter enzyme degrades kynurenine, a L-tryptophan metabolite, into immunologically inert, non-toxic and readily cleared metabolites, inhibiting tumor growth [14]. In addition, L-asparaginase also has being approved, and it is being used for the treatment of acute lymphoblastic leukemia using the same strategy described before: amino acid deprivation by enzymes [16,59].

Tumor lysis syndrome (TLS) is a health issue that may occur during cancer treatment, in which large amounts of tumor cells are lysed, releasing their contents into the bloodstream. As a consequence, hyperuricemia, an excess of uric acid, can emerge. The enzyme urate oxidase, that catalyzes the oxidation of uric acid to 5-hydroxyisourate, and its recombinant version, rasburicase, are being used to treat TLS [60]. Hyperuricemia triggered by other conditions, such as gout, can be also treated with this enzyme.

### 3.6. Cardiovascular Diseases

Cardiovascular disease (CVD) is the most common cause of death in the world. ERT is considered to treat this severe condition. First, urokinase is an enzyme whose substrate is plasminogen, an inactive form of the serine protease plasmin. This enzyme converts plasminogen to plasmin, which triggers a proteolytic cascade that participates in thrombolysis involving the degradation of the extra-cellular matrix (ECM). This process can be helpful in treating several vascular diseases [61]. Second, the enzyme nattokinase acts by inactivating plasminogen activator inhibitor 1, promoting fibrinolytic activity [17]. A compendium of CVD enzyme treatments is shown in Table 1.

### 3.7. Extracellular Matrix Disorders

There are some types of conditions in which a remodeling of the ECM is needed to recover its normal architecture. Matrix metalloproteinases play a key role in this process [62].

Healing involves several dynamic physiological processes, such as coagulation, tissue formation, re-epithelialization and ECM remodeling. During burn healing, native and denatured collagen in necrotic tissue need to be removed. In this framework, using collagenases, in particular CCH, can help to heal the wound and to minimize pain without increasing the risk of infection [63,64] (Table 1).

One of the main causes of cellulite is the accumulation of subdermal collagen in the dermal septa. Collagenase mixture injections have overcome phase III of clinical trial (NCT03446781) for cellulite treatment [65] (Table 1).

### 3.8. Reactive Oxygen Species Damage

Reactive oxygen species (ROS) are responsible for different types of DNA damage. ROS interaction with DNA can lead to mutations that affect its structure and function, triggering diverse pathologies.

Furthermore, ROS contribute to multiple organ failure in hemorrhagic shock. Superoxide dismutase enzyme could be a novel candidate to treat this pathology, as it catalyzes the dismutation of the superoxide radical into oxygen and hydrogen peroxide [66] (Table 1).

One of the alterations found in Parkinson’s disease (PD) is related to the change in mitochondria morphology by the abnormal α-synuclein, increasing superoxide formation [67]. Prion-like spreading and biocompatible antioxidant nanozyme (PtCu nanoalloys) could significantly inhibit α-synuclein pathology, cell death and neuron-to-neuron transmission by scavenging ROS [68] (Table 1).

### 3.9. Other Applications

Moreover, enzymes are used for many other different clinical approaches in addition to the ones above mentioned. For example, gluten-degrading peptidases are used in some cases for celiac diseases [69]. Matrix-degrading enzymes are used to degrade components in microbial biofilm and in cases of infections [70]. Furthermore, different proteolytic enzymes are studied to act against inflammation [71,72]. Notably, human butyrylcholinesterase (BChE) and bacterial cocaine esterase (CocE) are under study as novel therapies for cocaine overdose in animal models [73] (Table 1).

## 4. Current Challenges of Enzyme Therapies

Despite recent improvements and potential applicability of enzyme therapies, only a few of them have been approved by FDA and EMA. This phenomenon can be explained by mentioning the limitations of such approaches: short in vivo half-life, lack of tissue specificity and immunogenicity.

The administration of a molecule leads to multiple interactions that might cause a rapid loss of function or degradation of the enzyme [74]. However, in many circumstances, a fast clearance of the enzyme could be beneficial, in particular when the desired action has a limited time window, as in cases of cocaine overdose or in the process of wound healing. Regardless of its therapeutic approach, the application of an effective ERT to treat a metabolic deficiency needs to deal with the rapid clearance and degradation of enzymes that occurs upon administration in vivo. For instance, Fabry’s disease patients treated with recombinant human α-galactosidase A showed a rapid clearance of the enzyme. In a phase I/II clinical trial, a decrease of α-galactosidase A circulating concentration was observed due to a rapid elimination phase 1–2 hours after the infusion [75]. Furthermore, the high catalytic activity of enzymes represents considerable advantage but also a limitation. Regarding its limitation, enzymes do not usually distinguish between normal and pathologic tissue substrates and, consequently, might exhibit off-target interactions that can lead to toxic side effects [64]. In mucopolysaccharidoses pathologies (such as Hurler’s, Hunter’s, Morquio, Maroteaux-Lamy and Sly syndromes), ocular manifestations are common and may result in significant visual impairment due to corneal opacification, retinopathy, optic nerve swelling and atrophy, ocular hypertension, and glaucoma [76]. Additionally, due to toxic off-target effects, degeneration of the retina and abnormalities of the optic nerve have been observed in Hunter’s syndrome patients treated with ERT [77].

One of the main issues with enzyme-based therapies is patient immune response. The administration of an exogenous recombinant enzyme can trigger an immune response because the administered molecule itself becomes an immunogenic neo-antigen. In many immunogenicity studies of ERT in LSD, variable antibody responses have been observed. In Gaucher’s disease, 13% of the patients treated with glucocerebrosidase showed an immune response against the enzyme [78]. However, in the case of Hurler’s syndrome, an immune response against α-L-iduronidase has been observed in 50% of the patients [79]. Furthermore, 66% of patients affected by Pompe’s disease under study developed antibody titers to the infused α-glucosidase [80]. Lastly, in a study of Fabry’s disease, 88% of the patients generated anti-drug antibodies upon administration of recombinant α-galactosidase A [81]. Firstly, the onset of this response may drastically reduce the therapeutic efficacy, either by altering the pharmacodynamic interaction between the therapeutic protein and its target or by interfering with its pharmacokinetic profile. Thus, anti-drug antibodies may bind close to the enzyme binding or catalytic site, inducing a decrease in or loss of enzyme activity due to conformational changes or blocking the access of substrates. Furthermore, an increased clearance of the drug may be a consequence of the effect of the anti-enzyme antibodies, facilitating the action of professional antigen-presenting cells on therapeutic enzymes and thereby enhancing the immune response [82]. Secondly, the innate and adaptative immune responses may generate severe acute (e.g., anaphylaxis) and long-term medical conditions effects involving T-cell activation and innate immune responses including possible acute immune effects [83,84].

Many factors influence the immune response reaction against treatments based on enzymes. Genetic variations in the major histocompatibility complex, T-cell receptors or cytokines may alter the type and intensity of the immune response. Likewise, the patient’s age affects the immunogenicity of exogenous enzymes. In fact, elderly populations show milder immune responses to exogenous enzymes, as well as patients under immunosuppressive treatments and patients suffering pathologies of the immune system [85,86]. Additionally, individuals suffering an illness that activates the immune system, such as allergy or inflammation, could be predisposed to develop more potent immune reactions [87]. The route of enzyme administration also influences patient immune response. Thus, intravenous treatments tend to be less immunogenic than subcutaneous, intramuscular, mucosal or intradermal enzyme administrations [88]. Furthermore, long treatments and repeated exposure to enzymes after a long treatment-free period elicit more potent immune responses than short-term therapies. All the aforementioned phenomena may be also enhanced in patients having endogenous anti-enzyme cross-reactive antibodies before treatment with enzyme drugs [89]. On the other hand, the intrinsic immunogenicity of therapeutic enzymes could induce toxic effects. Acute reactions are typically developed a few hours after administration and can be IgE-mediated (typical anaphylactic reactions) or not [90]. Their symptoms include hypotension, bronchospasm, laryngeal or pharyngeal edema, wheezing and urticaria, being particularly severe in people with pre-existing cross-reactivity [89]. T-cell-dependent inflammatory responses are typically associated with symptoms that include fever, rash, myalgia, arthralgia and itching. Overall, the intrinsic immunogenicity of therapeutic enzymes is associated with a risk of triggering autoimmune diseases in susceptible patients.

## 5. Enzyme Therapies Troubleshooting

Enzymes have been used as therapeutic drugs for diverse pathologies [53,91,92]. Advances in both biotechnology and protein engineering have shed light on the study of enzymes’ potential as therapeutic tools and on the metabolic pathways involved in different diseases [93]. As a result, recombinant enzymes have emerged as new treatments for many diseases such as genetic abnormalities (LSD, CF, et cetera) and cancer, among other medical applications [93,94].

To become widely used drugs, enzyme therapies must overcome enzyme rapid clearance in vivo, the unwanted off-target interactions and patient immune response. The encapsulation and molecular modifications of enzymes, together with active monitoring of immune response, are the most remarkable therapy improvement techniques addressed to date.

One of the easiest ways to prevent unwanted off-target interactions is to directly apply the enzyme drug in the targeted tissue. In this context, urokinase has been applied via catheter to lysate intraluminal clots [95], and deoxyribonuclease has been administrated using eye drops for patients with dry eye disease [96]. However, different approaches are being developed for overcoming the detailed drawbacks, such as enzyme encapsulation and modification, as well as monitorization of patients’ immune responses.

### 5.1. Encapsulation of Enzymes

Enzyme encapsulation has been employed to transport the enzyme cargo in a more precise manner, improving target specificity and reducing immunogenicity and clearance [93,97]. Consequently, significant reductions in dose levels, off-target interactions and toxicity have been achieved [98,99]. Some examples for encapsulation vehicles are nanoparticles (NPs), virosomes, liposomes, extracellular vesicles (EVs) and erythrocytes. On one hand, NPs, both biological (usually lipid-based) and inorganic (silica NPs, quantum dots, gold NPs, iron oxide NPs, et cetera), are multifunctional scaffolds with properties which augment their role as delivery vehicles. NPs take advantage of their structural, chemical, mechanical, magnetic, electrical and biological properties that allow a precise and controlled release of drugs [100]. For example, NPs containing pyruvate dehydrogenase are being studied as a therapy for *Pseudomonas aeruginosa* biofilm-associated infections [101]. One interesting type of NPs are vault-derived nanoparticles. Vaults are naturally occurring human intracellular ribonucleoprotein particle complexes, which form large barrel-shaped hollow nanocapsules. For instance, manganese peroxidase has been encapsulated in vault NPs and is being studied for biodegradation of organic contaminants [102]. Enzymes can be encapsulated within these structures, leading to enhanced stability [102,103] and, when coupled with target-directing molecules such as monoclonal antibodies, can be delivered efficiently to the desired region [104]. On the other hand, virosomes are produced based on some features from viruses to improve the delivery of drugs during enzyme treatments. Virosomes, like viruses, bind to and enter the cytosol of specific cell types. Their major limitation is the patient immune response upon exposure to virosomes [105]. Until now, virosomes have not been used for enzyme delivery, but they have interesting potential as vehicles that has been demonstrated for anti-cancer drug delivery, antigen delivery and adjuvant delivery for vaccines [105]. Liposomes are lipid vesicles with one or more bilayers. They are widely used as delivery platforms due to their ability to enter the cytoplasm [106]. For example, liposomes are being studied for the delivery of palmitoyl-protein thioesterase-1 in infantile neural ceroid lipofuscinosis, leading to restored levels of enzymatic activity in patients’ fibroblasts [107]. EVs are proteoliposomes released from the cell membrane that act similarly to synthetic liposomes, offering interesting characteristics [108]. EVs are being studied in vivo for the delivery of catalytic enzymes. Cre recombinase and β-lactamase have been loaded and delivered in EVs’ known as gectosomes, which are programmable, highly fusogenic vesicles [109]. Lastly, erythrocytes are being used as drug delivery systems thanks to their low immunogenicity, the long in vivo circulation time due to a reduced clearance, the theoretical unnecessity of developing chemical modifications of the enzyme, and the protection offered by the membrane, allowing the enzyme to remain active. Enzymes can be coupled to the erythrocyte membrane; for instance, in in vivo studies, tissue plasminogen activator was coupled to the external red-cell membrane, improving its fibrinolytic profile [110]. On the other hand, the enzyme can be encapsulated in the erythrocyte. Many publications have reviewed the current usages of erythrocytes as enzyme delivery vehicles following this strategy [111,112]. As some examples, erythrocyte-containing asparaginase (eryaspase) is showing promising results in phase III clinical trials as a treatment for different cancers when combined with chemotherapy. Other enzymes such as arginine deiminase or methionine gamma lyase are being studied for cancer therapy when coupled with erythrocytes. Phenylalanine ammonia lyase (PAL) is approved as a therapeutic alternative for ERT in PKU, and the encapsulation of this enzyme in erythrocytes is being studied as a good strategy to overcome the drawbacks of the current ERT treatment. Additionally, erythrocytes are being used in mitochondrial neurogastrointestinal encephalomyopathy (MNGIE) to compensate for the deficiency in thymidine phosphorylase by delivering the enzyme. The status of Orphan Drug was provided by both FDA and EMA for erythrocyte encapsulated thymidine phosphorylase, and phase II clinical trials are under development. Furthermore, erythrocytes containing alcohol oxidase are undergoing promising preclinical studies for alcohol detoxification [111,112]. Two companies are leading the innovations in this area: EryDel in Italy, and Erytech in France. EryDel focuses on encapsulating small and large molecules, including therapeutic enzymes, in patients’ red blood cells. This company is carrying out phase III clinical trials with erythrocyte coupled with thymidine phosphorylase, as well as preclinical studies with other enzymes coupled with red blood cells, such as PAL for PKU, uricase for refractory gout, guanidinoacetate N-methyltransferase (GAM) for GAM deficiency and cocaine esterase for cocaine addiction. In turn, Erytech uses allogenic erythrocytes as vehicles. This company focuses principally on cancer therapy, and its leading drug is eryaspase for the treatment of different tumors. Despite the promising results of using erythrocytes as vehicles for enzyme delivery, some drawbacks should be considered. First, when using allogenic red blood cells, the problems of transfusing blood products arise, such as rejection or transmission of infections, among others. In addition, the production of cell products requires intense sterile work, and the large scale of the production makes it difficult. If the quality of the erythrocytes is not high enough, they can degrade when administrated, releasing the enzyme uncontrollably and producing toxic side effects. Furthermore, low-molecular-weight compounds easily pass through the cell membrane, leaving the erythrocyte and making it difficult to create long-term deposits of the enzyme. To overcome this, the enzyme can be modified to slow the release, but an activation change should be performed inside the cell, causing a variable response among patients, which prevents stable results from being obtained. Alternatively, the membrane of the erythrocyte can be modified, but these changes make it more recognizable by reticuloendothelial system cells, being quickly removed from the bloodstream. Additionally, specific transporters, shuttles or endocytosis, as well as exocytosis processes, can be used to overcome this problem. Thus, there are some disadvantages to the use of erythrocytes as drug carriers that call for further improvements to experimental methods [113].

### 5.2. Modification of Enzymes

The chemical modification of enzymes offers alternatives to improve their therapeutic properties. Some examples of targeting agents that are conjugated with enzymes are antibodies and biomolecules such as proteins, peptides, saccharides, hormones, vitamins, DNA [114,115] and protein–polymer conjugates, such as PEG. PEG is a nontoxic, nonimmunogenic and amphipathic polymer widely used to modulate the activity and pharmacokinetics of enzyme drugs, affecting the immunoreactivity, immunogenicity and in vivo degradation of the enzymes [116]. PEG-aspargase (Oncaspar) is a PEGylated form of native *Escherichia coli*-derived L-asparaginase, which is known in the USA and Europe as an effective treatment for acute lymphoblastic leukemia. Compared to non-PEGylated L-asparaginase, PEG-aspargase presents prolonged circulation times caused by the reduced clearance of the enzyme, leading to less frequent administrations. Additionally, PEG modifications show a reduction in the immunogenicity of the enzyme, resulting in a better tolerability profile [117]. Furthermore, DNA is also being studied to create nanocage vehicles that can respond to stimuli such as pH, ligands and temperature depending on their sequences [118,119,120]. In many cases, the molecule modifications can produce main problems such as reduction of stability, as well as the mitigation of immune reactions [121].

### 5.3. Monitorization of Patients’ Immunoresponses

The effectiveness of enzymatic treatments depends not only on their in vivo half-life and their tissue specificity, but also on the drug-induced immune response of the patient. The development of anti-drug antibodies (mostly immunoglobulins G, IgG, and M, IgM) can compromise therapy effectiveness and individual safety. Thus, a great effort is being made to develop quantitative methods to monitor specific biomarkers related to the immunological responses and inflammation associated with the disease [24,122]. Additionally, monitoring the immune system may be useful for assessing clinical risks associated with therapeutic enzymes. The evaluation of immunogenicity starts with screening assays to detect clinically relevant antibodies such as IgG or IgM. Then, a confirmation of the presence of these antibodies should be performed followed by a neutralization assay, in which the capacity of said antibodies to avoid substrate processing is evaluated. Finally, it is important to assess the immunogenicity of enzymatic treatments, in order to prevent fatal reactions and development of autoimmunity [89]. Enzyme-linked immunosorbent assays (ELISA), in which antibodies are immobilized on solid surfaces, are currently the gold standard for studying biomarkers. However, these immunoassays are labor-intensive, require specific facilities and personnel, and show high false positive rates for some biomarkers. Consequently, ELISAs are usually complemented with other studies such as bridging assays, plasmonic technology or electroluminescence-based techniques to improve the quality of measurements. In order to overcome some of these limitations and to improve the sensitivity, new approaches such as microarray technologies are reaching new heights for the quantification of biomarkers [123].

Microarray technology allows the immobilization of cell membranes, antibodies, enzymes and other proteins, as well as whole cells, on different surfaces without disrupting their functional activity. Therefore, they are very versatile tools for immunochemistry, autoradiography, radioligand-binding studies, mitochondrial toxicity assays [124,125,126] or other approaches such as colorimetric and mass spectrometry techniques [126,127]. Microarrays allow a reduction of the number of samples, drugs, chemicals and radioactive residues. In this regard, microarray technology has also been previously applied for the detection of biomarkers in the diagnosis of disease, in particular, the inflammation protein MMP9 in dry eye disease [128]. Microarrays show greater sensitivity for the detection of these biomarkers than conventional standard ELISA, justifying the superiority of this technique for clinical settings [123]. Microarrays also offer a robust and cost-effective alternative for the development of screening assays to detect IgG and IgM, and they are being used for severe acute respiratory syndrome coronavirus 2 (SARS-CoV-2) infection diagnosis [129]. Additionally, microarrays are being used for confirming and profiling antibody responses to microbial infections [130,131]. Safety of treatment can be also analyzed using microarrays to study patient autoimmunity [132]. In this sense, antigen-reactive antibody profiles can be investigated using proteome microarrays, allowing immune responses of patients to be assessed [133].

**Figure 2:**
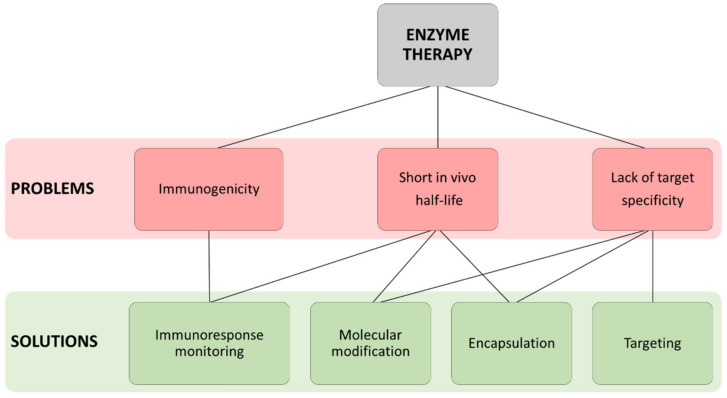
Outline of the main problems presented when using enzyme therapies as well as the different solutions applied to overcome them.

In summary, new strategies are being developed in the field of therapy with enzymes. Both encapsulation techniques and the molecular modification of enzymes have been shown to improve the effectiveness of treatments. Moreover, improved monitoring of immune response against therapeutic enzymes has contributed to a better management of clinical symptoms. This monitorization can now be addressed with new methods, in addition to conventional ELISA, as commercially available microarrays show higher sensitivity and provide a higher multiplexing capability (Figure 2).

## 6. Future Perspectives

The high applicability of enzymes for treating different conditions, as well as the advances that are being made to overcome their associated issues, has led to a steady increase in the use of these treatments, which will be even greater in the future. Innovative biotechnology strategies are being developed to solve enzyme drugs drawbacks; NPs are being studied as vehicles [100], as well as virosomes [105], liposomes [106], EVs [108] and erythrocytes [113]. Additionally, molecular modifications are being carried out to improve enzyme characteristics; conjugations with biomolecules such as antibodies, DNA or metabolites are under study [114,115,118,119], and PEG modifications are being used in therapy [116]. At the same time, novel approaches to enzyme therapy applications are under study for cancer [14,15,16,60], neurodegenerative diseases [68], joint problems [56,57,58], inflammation [71,72] and infections [70].

The use of enzymes as therapeutics has also contributed to the treatment of COVID-19, caused by SARS-CoV-2. To infect a cell, SARS-CoV-2 binds to the angiotensin-converting enzyme 2 (ACE2) at the cell surface. Binding of ACE2 triggers a conformational change in the spike protein of the virion, exposing protease-sensitive peptides, which upon cleavage, lead to internalization of the virus and infection [134]. ACE2 is involved in the regulation of the renin–angiotensin system, and is mainly associated with vasodilation, anti-inflammatory and anti-fibrotic functions [135]. These important functions, together with the binding capacity of SARS-CoV-2, have led to the development of human recombinant soluble ACE2 (hrsACE2) for the treatment of COVID-19. hrsACE2, also referred as APN01 (Apeiron Biologics, Vienna, Austria), has been shown not only to prevent virus entry but also to downregulate inflammation without impairing antibody production [136,137,138,139]. APN01 has completed successfully phase II of clinical trials (NCT04335136), offering a promising alternative to the already existing treatments. APN01 prevents virus uptake, but once the virus has entered into the cell, RNases form a defense mechanism against single-stranded RNA viruses, posing as attractive tools for virus therapies [140]. From these, the binase from *Bacillus pumilus* decreased both MERS-CoV and HCoV-229E viral loads in the context of infection [141]; however, its efficacy against SARS-CoV-2 remains to be studied.

COVID-19 is often referred to as a dysregulation of the inflammatory response, provoked by an increased release of ROS, cytokines and chemokines, which prompts tissue damage and might lead to the death of the individual. Approaches using a modified formulation of the catalase, an ubiquitous antioxidant enzyme, have proven to decrease viral load and enhance recovery when administrated intravenously or nebulized in rhesus macaques [142], attractive qualities which make it suited for human studies. Moreover, an in silico study has suggested attractive proteases as therapeutics against SARS-CoV-2 [143]. This study addressed the binding capabilities of fungal proteases, some of which are used in the food and textile industries, to several of the SARS-CoV-2 proteins. These proteases have been hypothesized to bind and inactivate or even degrade virions, offering attractive candidates for future considerations in COVID-19 therapeutics.

Viruses are rapidly evolving pathogens, allowing them to adapt quickly to new environments. Monotherapy often leads to the development of resistance, treatment failure and spread of new variants [144]. The limited supply of post-exposure COVID-19 therapeutics complicates the use of drug combinations in treatments. Enzymes as therapeutics are not only showing promising results in monotherapy but also have already been tested cooperatively with remdesivir, improving patient recovery while minimizing the risk of viral escape [145].

## 7. Conclusions

In conclusion, enzyme therapy is an emerging strategy for treatment of a wide range of pathologies such as metabolic disorders, fibrosis, cancer, CVD and SARS-CoV-2 infections, among others. However, the short functional in vivo half-lives of therapeutic enzymes due to their exposure to endogenous degrading mechanisms, unwanted adverse effects and toxicity, poor tissue specificity, as well as the activation of immune responses, must be improved to develop its therapeutic potential. Thanks to the advances in the biotechnology field, these limitations are being overcome. Enzyme encapsulation approaches, such as liposomes, membrane vesicles, nanoparticles and erythrocytes, improve in vivo half-life, tissue specificity and reduce immunogenicity of enzymes. Targeted enzyme modification technology, such as PEG conjugation, also results in an improvement of functional bioavailability and reduced immunogenicity. Lastly, the monitoring of patients’ immune responses may significantly improve patient management to preserve efficacy and safety of therapy. In this context, microarray technology is emerging as a practical tool to improve monitoring of anti-enzyme immune responses in patients treated with ERT. Considering the great therapeutic potential of enzymes as drugs, further research is still needed to broaden their applicability to a wider spectrum of diseases.

## Figures and Tables

**Table 1:** Summary of the main pathologies and conditions treated with enzymes.

Disease/ Condition	Cause/Pathology	Therapeutic Enzymes *	Ref.
Lysosomal storage diseases			
Gaucher’s disease	Deficiency of glucocerebrosidase	Glucocerebrosidase [Cerezyme, Vprip, Taliglucerase alpha]	[25], (a,b,c)
Hunter’s syndrome	Deficiency of iduronate-2-sulfatase	Iduronate-2-sulfatase [Elaprase]	[26], (d)
Fabry’s disease	Deficiency of α-galactosidase A	α, β-galactosidase A [Replagal, Fabrazyme]	[28], (e,f)
Hurler’s syndrome	Deficiency of α-L-iduronidase	α-L-iduronidase [Aldurazyme]	[29], (g)
Morquio syndrome type A	Deficiency of N-acetylgalactosamine-6-sulfate sulfatase	N-acetylgalactosamine-6-sulfate sulfatase [Vimizim]	[30], (h)
Maroteaux-Lamy syndrome	Deficiency of arylsulfatase B	N-acetylgalactosamine-4-sulfatase [Naglazyme]	[31], (i)
Sly syndrome	Deficiency of β-glucuronidase	β-glucuronidase [Mepsevii]	[32], (j)
α-Mannosidosis	Deficiency of α-D-mannosidase	Velmanase α [Lamzede]	[33], (k)
Batten disease	Deficiency of tripeptidyl peptidase 1	Cerliponase α [Brineura]	[34], (l)
Pompe’s disease	Deficiency of acid α-glucosidase	α-glucosidase [Myozyme]	[35], (m)
Metabolic deficiencies			
Exocrine pancreatic insufficiency (EPI)	Insufficient secretion of pancreatic enzymes	Pancreatic enzymes [Enzepi]	[36,37,38], (n)
Phenylketonuria (PKU)	Deficiency of phenylalanine hydroxylase (PAH)	PAH and phenylalanine ammonia-lyase PAH [Palynziq]	[39], (o)
Severe combined immunodeficiency (SCID)	Deficiency of adenosine deaminase (ADA)	Polyethylene glycol-conjugated ADA	[40,41]
Wolman disease	Deficiency of lysosomal acid lipase	Lysosomal acid lipase [Kanuma]	[42], (p)
Acute intermittent porphyria (AIP)	Deficiency of hydroxymethylbilane synthase	Hydroxymethylbilane synthase and porphobilinogen deaminase	[43]
Congenital sucrase-isomaltase deficiency (CSID)	Deficiency of sucrase and isomaltase	Sacrosidase	[44]
Hypophosphatasia	Deficiency of tissue-nonspecific isoenzyme of alkaline phosphatase (TNSALP)	TNSALP [Strensiq]	[45], (q)
Protein C deficiency	Deficiency of Protein C	Protein C [Ceprotin]	[46], (r)
Lactose intolerance	Reduction or loss of the activity of lactase-phlorizin hydrolase	Lactase	[47]
Fibrosis conditions			
Chronic total occlusions	Fibrous plaques obstructing coronary arteries	Collagenase *Clostridium histolyticum* (CCH)	[48]
Dupuytren’s disease	Thickening of the fascia tissue in the hands	Collagenase *Clostridium histolyticum* (CCH) [Xiapex]	[22,49], (s)
Peyronie’s disease	Fibrous plaques formation in the penis	Collagenase *Clostridium histolyticum* (CCH)	[20]
Uterine fibroid	Fibroid tissue growth around the uterus	Collagenase *Clostridium histolyticum* (CCH)	[50]
Keloid disease	Overgrowth of granulation scar tissue	Collagenases and matrix metallopeptidases	[51,52]
Lung cystic fibrosis	Viscose secretions in the lungs	Deoxyribonuclease I [Pulmozyme]	[21], (t)
Glaucoma	Fibrous formations at the trabecular meshwork of the eye	Collagenases	[54]
Ocular affections			
Different ocular diseases treated with vitrectomy	Malfunction of the vitreous humor of the eye solved by its enzymatic removal	Chondroitinase, hyaluronidase, nattokinase and ocriplasmin [Jetrea]	[55], (u)
Joint problems			
Intervertebral disc herniation	Disc material penetrating the spinal dura	Chondroitin sulfate ABC endolyase	[56]
Arthritis	Osteophytes formation and inflammation	Proteolytic enzymes	[57,58]
Cancer			
Different types of cancer	Increased amino acid metabolism in the tumor microenvironment	PEGylated arginine deaminase and kynureninase [Voraxaze, PEG hyaluronidase PH20]	[14,59], (v,w,x)
Leukemia	Increased amino acid metabolism in the tumor microenvironment	L-asparaginase [Spectrila, Kidrolase, Erwinase, Oncaspar]	[16,59], (y,z)
Chemotherapy-induced hyperuricemia	Increase in uric acid due to tumor lysis syndrome	Urate oxidase and rasburicase [Fasturtec]	[60], (aa)
Cardiovascular diseases			
Cardiovascular disease	Formation of fibrin clots degraded by plasmin	Nattokinase and urokinase [Streptase, Syner-Kinase, Kinclytic, Rapilsyn, Actilyse, Metalyse]	[17], (ab,ac,ad,ae,af)
Extracellular matrix disorders			
Burns	Denatured collagen in necrotic tissue	Collagenase *Clostridium histolyticum* (CCH) [Nexobrid]	[63,64], (ag)
Cellulite	Accumulation of subdermal collagen in the dermal septa	Collagenases	[65]
Reactive oxygen species damage			
Organ injury in hemorrhagic shock	Reactive oxygen species (ROS) tissue damage	Superoxide dismutase	[66]
Parkinson’s	Reactive oxygen species (ROS) tissue damage	Nanozyme (PtCu nanoalloys)	[68]
Other applications			
Celiac disease	Gluten intolerance	Gluten-degrading enzymes	[69]
Microbial infections	Microbial biofilm formation during infection	Matrix-degrading enzymes (polysaccharide-degrading enzymes, nucleases and proteases)	[70]
Inflammation	Inflammation of overexpressed pathways disrupting physiological homeostasis	Proteolytic enzymes (trypsin or serratiopeptidase)	[71,72]
Cocaine overdose	Cocaine toxicity	Human butyrylcholinesterase (BChE) or Bacterial cocaine esterase (CocE)	[73]

* Tradenames of the enzymes are given in brackets. Lowercase letters reference to enzyme drugs authorized by the EMA, a list of them is detailed in Appendix A.

## Data Availability

Not applicable.

## Appendix A

List of enzyme drugs authorized by the EMA, the first code es the ATC.

- A09AA02 Cerezyme (Imiglucerase) 1997.
- A16AB10 Vprip (Velaglucerase alpha) 2010.
- A16AB11 Taliglucerase alpha (glucocerebrosidase) 2010.
- A16AB09 Elaprase (Idursulfase) 2007.
- A16AB04 Fabrazyme (Agalsidase beta) 2001.
- fA16AB03 Replagal (Agalsidase alpha) 2001.
- A16AB05 Aldurazyme (Lanoridase) 2003.
- A16AB12 Vimizim (Elosulfase alpha) 2014.
- A16AB08 Naglazyme (Galsulfase) 2006.
- A16AB18 Mepsevii (Vestronidase Alfa) 2018.
- A16AB15 Lamzede (Velmanase alpha) 2018.
- A16AB17 Brineura (Cerliponase alpha) 2017.
- A16AB07 Myozyme (Alglucosidase alpha) 2006.
- A09AA02 Enzepi (Multienzymes) 2016.
- A16AB19 Palynziq (PEGvaliace) 2010.
- A16AB14 Kanuma (Sevelipase alpha) 2010.
- A16AB13 Strensiq (Arfotase alpha) 2015.
- B01AD12 Ceprotin (Protein C) 2001.
- M09AB02 Xiapex (Collagenase Clostridium Histolyticum) 2011.
- R05CB13 Pulmozyme (Dornase alpha) 2017.
- S01XA22 Jetrea (Ocriplasmin) 2013.
- - Voraxaze (Carboxypeptidase G2) 2003.
- B06AA03 PEG hyaluronidase PH20 (pegvorhyaluronidase alpha) 2014.
- - PEGarginine deaminase 2005.
- L01XX02 Spectrila, Kidrolase, Erwinase (L-asparaginase) 2016.
- L01XX24 Oncaspar (PEGasparginase) 2016.
- V03AF07 Fasturtec (Rasburicase) 2001.
- B01AD01 Streptase (Streptokinase) 1960.
- B01AD04 Syner-Kinase, Kinclytic (Urokinase) 2019.
- B01AD07 Rapilsyn (Reteplase) 1996.
- B01AD02 Actilyse (Alteplase) 1999.
- B01AD11 Metalyse (Tenecteplase) 2001.
- D03BA03 // M09AB03 NexoBrid (Proteolytic enzymes enriched in bromelain) 2012.
